# Immediate Effect of Whole Body Vibration on Knee Extensor Tendon Stiffness in Hemiparetic Stroke Patients

**DOI:** 10.3390/medicina57101037

**Published:** 2021-09-29

**Authors:** Shih-Ting Tsai, Cyuan-Fong Li, Kai-Chiao Chi, Li-Wei Ko, Cory Stevenson, Yi-Jen Chen, Chia-Hsin Chen

**Affiliations:** 1 Department of Physical Medicine and Rehabilitation, Kaohsiung Medical University Hospital, Kaohsiung 807, Taiwan; alisontsai0329@gmail.com (S.-T.T.); alee0425@gmail.com (C.-F.L.); otchi626@gmail.com (K.-C.C.); 2 Department of Physical Medicine and Rehabilitation, Kaohsiung Municipal Siaogang Hospital, Kaohsiung 812, Taiwan; 3 Institute of Bioinformatics and Systems Biology, National Yang Ming Chiao Tung University, Hsinchu 30010, Taiwan; lwko@nctu.edu.tw (L.-W.K.); cesteven@nctu.edu.tw (C.S.); 4 Center for Intelligent Drug Systems and Smart Bio-Devices (IDS2B), National Yang Ming Chiao Tung University, Hsinchu 30010, Taiwan; 5 Drug Development and Value Creation Research Center, Kaohsiung Medical University, Kaohsiung 807, Taiwan; 6 Department of Physical Medicine and Rehabilitation, School of Medicine, College of Medicine, Kaohsiung Medical University, Kaohsiung 807, Taiwan; 7 Regenerative Medicine and Cell Therapy Research Center, Kaohsiung Medical University, Kaohsiung 807, Taiwan; 8 Department of Physical Medicine and Rehabilitation, Kaohsiung Municipal Ta-Tung Hospital, Kaohsiung 801, Taiwan

**Keywords:** stroke, tendon stiffness, whole body vibration, rehabilitation, elastography

## Abstract

*Background and Objectives:* Whole body vibration is widely used to enhance muscle performance, but evidence of its effects on the tendon stiffness of the knee extensor tendon in stroke remains inconclusive. Our study was aimed to determine the difference in patellar and quadriceps tendon stiffness between hemiparetic and unaffected limbs in stroke patients and to investigate the immediate effect of whole body vibration on tendon stiffness. *Materials and Methods:* The patellar and quadriceps tendon stiffness of first-ever hemiplegic stroke patients was evaluated with elastography to compare the differences between hemiparetic and unaffected limbs. After one 20 min session of whole body vibration exercise in the standing position, tendon stiffness was again measured to evaluate the immediate effects of whole body vibration on tendon stiffness. *Results:* The results showed no significant differences in the tendon stiffness of the patellar and quadriceps tendons between hemiparetic and unaffected limbs. However, significant associations were found between the tendon stiffness of the patellar and quadriceps tendons and knee extensor spasticity on the hemiparetic side (ρ = 0.62; *p* = 0.044). There were no significant changes in tendon stiffness after a single session of whole body vibration. *Conclusions:* In conclusion, knee extensor tendon stiffness in hemiparetic limbs is positively correlated to the degree of knee extensor spasticity in stroke patients. However, a single session of whole body vibration does not alter tendon stiffness.

## 1. Introduction

Decreased tendon stiffness has been noted in older people and paralytic patients due to spinal cord injury [1,2]. The main mechanism of change in the mechanical properties of tendons is the adaptation to reduced loading, which is related to decreased synthesis in loading-induced collagen [2,3]. Moreover, decreased tendon stiffness in the elderly is reversible with regular loading from strength training, and this improvement is dependent on load magnitude [4,5].

Weakness is one of the cardinal symptoms of patients with stroke. A study by Hunnicutt et al. showed that the knee extensor strength of hemiplegic limbs was 36% lower than that of nonparetic limbs [6]. Moreover, in patients with chronic stroke, the muscle volume of the quadriceps was reported to be 24% reduced than that of nonparetic limbs [7]. The biomechanical and physiological changes in knee extensors after stroke may lead to an unloading effect on tendons that is similar to the effects found in spinal cord injury patients and the elderly.

Whole body vibration (WBV) has been applied to improve muscles strength, power, and flexibility [8]. However, there have been limited reports regarding the effect of vibration on tendon stiffness, with inconsistent results [9,10,11,12,13]. Shear wave elastography is a commonly used tool in the evaluation of the musculoskeletal system, and it considered to be a reliable method for the assessment of patellar and quadriceps tendon mechanical properties using shear wave velocity [14,15,16,17].

We hypothesized that unloading due to knee extensor weakness in a stroke population leads to decreased knee extensor tendon stiffness. The objectives of this study were to investigate changes in knee extensor tendon stiffness in a stroke population using shear wave elastography and to evaluate the immediate effect of WBV on the tendon stiffness of a hemiplegic stroke population.

## 2. Materials and Methods

### 2.1. Participants

This study was conducted on participants with first-ever hemiplegic stroke. The inclusion criteria were as follows: the subjects (1) have an age of 20 years or older, (2) have experienced their first hemiparetic stroke, (3) were more than three months from the onset of stroke, (4) were able to stand with or without a device, and (5) could cooperate with the evaluation. The exclusion criteria were as follows: the subjects could not have (1) experienced recurrent stroke, (2) experienced stroke onset within three months, (3) received knee surgery before, (4) a limited range of motion of the knee joint, (5) acute arthritis attack, (6) lower extremity fracture in recent six months, (7) weakness due to other neuromuscular diseases, (8) an inability to cooperate with the evaluation. The study was approved by the Institutional Review Board of our institute. All subjects were given and signed informed consent before participation.

### 2.2. Assessment and Whole Body Vibration Exercise

**Figure 1:**
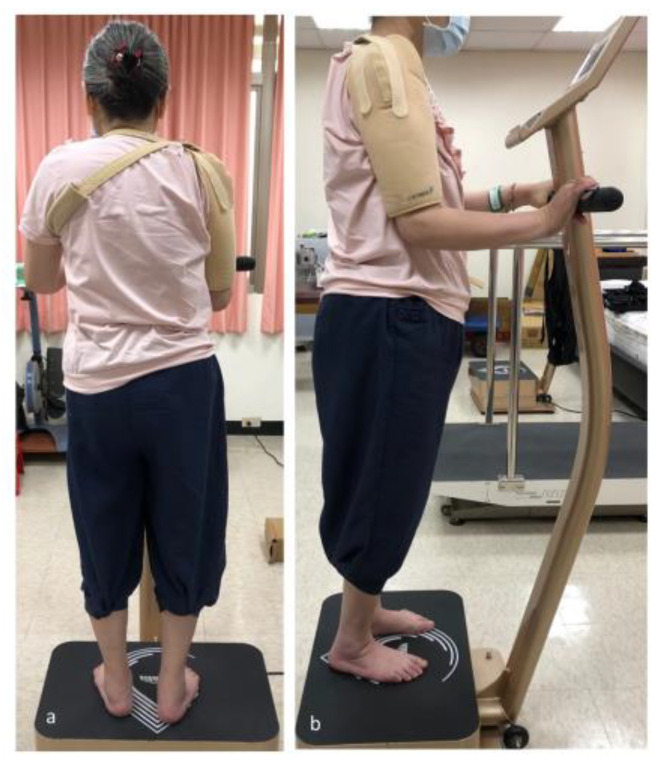
Whole body vibration exercise in the standing position.

The knee extensor spasticity of the hemiparetic limb of all participants was evaluated using the Modified Ashworth scale (MAS), which has six degrees (0, 1, 1+, 2, 3, and 4), and transformed to 0, 1, 2, 3, 4, and 5 for further statistical analysis. Knee sonography was performed to assess the thickness and mechanical properties (tendon stiffness and shear wave velocity) of the patellar and quadriceps tendons. Then, participants received 20 min of WBV exercise in the standing position (Figure 1), which was composed of two sessions of 10 min of vibration with a 3-minute resting interval. The tendon shear wave velocity was measured again immediately after WBV exercise.

### 2.3. Ultrasound Examination Protocol

The grayscale sonography and elastography of the patellar and quadriceps tendons were performed with an Acuson S2000 ultrasound system (Siemens Medical Solutions, Mountain View, CA, USA) equipped with 9L4 (4–9 MHz) linear array ultrasound transducer. The room temperature was controlled at 25 °C.

**Figure 2:**
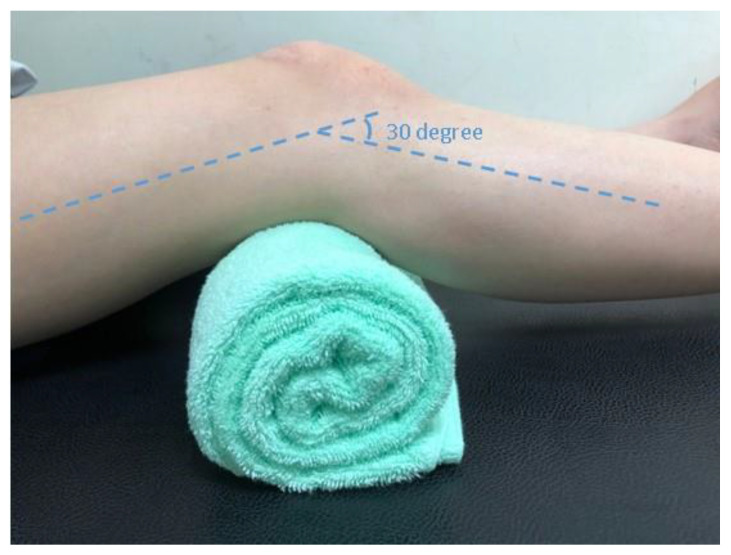
Knee positioned at 30 degrees of flexion for ultrasound evaluation.

**Figure 3:**
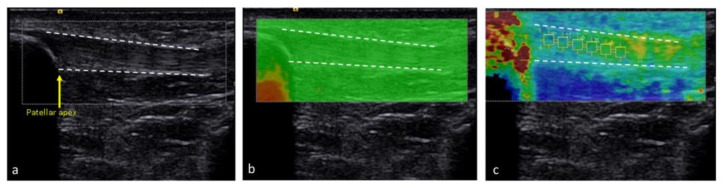
We used the B-mode to localize the patellar tendon (**a**) and then shifted to the elastography mode. The quality map of patellar tendon (**b**) was checked, and then we measured the shear wave velocity (**c**). Dashed line indicates patellar tendon border.

**Figure 4:**
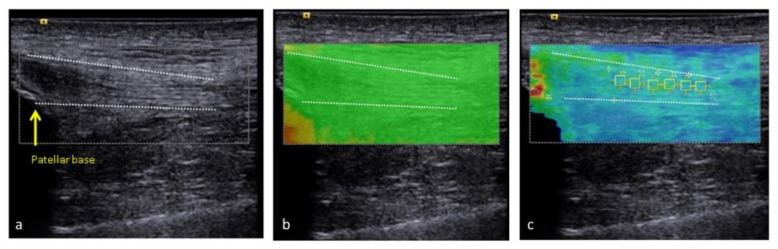
We used the B-mode to localize the quadriceps tendon (**a**) and then shifted to the elastography mode. The quality map of the quadriceps tendon (**b**) was checked, and then we measured the shear wave velocity (**c**). Dotted line indicates quadriceps tendon border.

Before testing, participants rested in the supine position for five minutes to ensure the mechanical properties of quadriceps and patellar tendons were evaluated at resting unloaded status. The measurement was performed with the knee flexed at 30 degrees (Figure 2). We used grayscale sonography first to localize the tendon and then measured its thickness. Patellar tendon thickness was measured by longitudinally placing the transducer and measuring the regions 10, 15, and 20 mm distal to the apex of the patella (Figure 3a). Quadriceps tendon thickness was measured at levels of 10, 15, and 20 mm proximal to the patellar base (Figure 4a).

Then, we shifted to elastography to assess tendon stiffness with shear wave velocity. Before measuring shear wave velocity, we checked the quality map to make sure there were no red areas that indicated poor quality in the region of interest (Figure 3b and Figure 4b). The measurement of patellar tendon stiffness was conducted on the area between 5 and 20 mm from the apex of the patella (Figure 3c). The measurement of quadriceps tendon stiffness was assessed on an area between 10 and 25 mm from the patella base (Figure 4c). The same measurements of tendon thickness and stiffness were repeated two times, and the average value of the two measurements was used for analysis.

### 2.4. Statistical Analysis

Descriptive data were reported as means and standard deviations. The tendon thickness and stiffness were analyzed by the Wilcoxon rank sum test for comparisons between hemiparetic and unaffected sides. The correlation between tendon thickness, stiffness, change after WBV, and knee extensor spasticity was analyzed using Spearman’s correlation. Tendon thickness and stiffness were analyzed with the Wilcoxon signed rank test for comparisons of before and after WBV exercise in hemiparetic and unaffected sides, as well as the change in mean differences of the hemiparetic and unaffected sides before and after WBV exercise. We used SAS (version 9.4; SAS Institute, Cary, NC, USA) for all analyses. A *p*-value < 0.05 was regarded as statistically significant.

## 3. Results

A total of 11 participants were enrolled. The basic sociodemographic data and clinical characteristics of the study population are shown in Table 1. The average age was 53.6 ± 4.4 years old, and the average time from onset of stroke was 9.91 ± 1.89 months. Eight patients had ischemic stroke, and three patients had hemorrhagic stroke.

No statistically significant differences between hemiparetic and unaffected sides were observed in the tendon thickness and stiffness of the patellar and quadriceps tendons at baseline measurement (Table 2). However, the stiffness values of the patellar and quadriceps tendons in the hemiparetic side were correlated to knee extensor spasticity (ρ = 0.62; *p* = 0.044) (Table 3).

After WBV exercise, no statistically significant change was observed in patellar and quadriceps tendon stiffness in the hemiparetic or unaffected sides. However, there were trends of decreased stiffness in the patellar tendon (mean change: −0.36; *p* > 0.05) and the quadriceps tendon (mean change: −0.38; *p* > 0.05) in the hemiparetic side. In the unaffected side, smaller changes in the stiffness of the patellar tendon (mean change: 0.00; *p* > 0.05) and the quadriceps tendon (mean change: −0.14; *p* > 0.05) were observed (Table 4).

The mean differences between the patellar and quadriceps tendon stiffness of the hemiparetic and unaffected sides were observed to be decreased after WBV exercise, although they did not reach statistically significant levels (mean change of −0.36 and *p* > 0.05 for the patellar tendon; mean change of −0.24 and *p* > 0.05 for the quadriceps tendon; Table 5).

The knee extensor spasticity was not correlated to tendon stiffness change after WBV exercise in the hemiparetic side (Table 6) or the change in tendon stiffness difference between the hemiparetic and unaffected sides after WBV exercise (Table 7).

## 4. Discussion

To the best of our knowledge, this is the first study to analyze the mechanical properties of the patellar and quadriceps tendons of the hemiparetic and unaffected limbs of individuals with stroke.

Patients with hemiplegic stroke have motor impairment, which leads to reduced loading over affected lower limbs. Previous research has shown lower Achilles tendon stiffness in the affected legs of patients with stroke [18,19,20,21]. However, there are no available reports concerning the change in tendon property in the knee extension mechanism, including patellar and quadriceps tendons, in stroke patients. Regarding morphological properties, some studies have shown smaller cross-section areas of the Achilles tendon in the affected side than that in the unaffected side [19,21]. Other studies have shown no difference of the Achilles tendon cross-section area between impaired and healthy side [18].

In this study, there were no significant differences in the tendon stiffness of the patellar and quadriceps tendons between hemiparetic and unaffected limbs. This finding was inconsistent with previous studies, which reported change in Achilles tendon stiffness in stroke patients [18,19,21]. However, the results showed significant positive correlation between knee extensor spasticity and tendon stiffness in both the patellar and quadriceps tendons. This suggested that despite a weaker knee extensor muscle due to hemiplegia, the tendon is still provided with sufficient loading stimulus so the integrity of knee extensor tendon can be preserved. Moreover, in stroke patients, in addition to weakness, other mechanisms can cause inefficient tension generation in large muscle groups, resulting in functional impairment including abnormal muscle tone and the poor coordination of muscle contraction [22]. In the knee joint, co-contraction in the knee extensor and knee flexor is a compensatory strategy for increasing knee stability but contributes to stiff knee gait [23,24]. The stiff knee gait is related to the hyperreflexia of the rectus femoris [25]. The results in our study were in line with the fact that a large portion of stroke patients in this study were able to walk with an assistive device. Moreover, the involuntary muscle contraction from an increasing stretching reflex due to spasticity in the affected leg may help to maintain the mechanical loading of the hemiparetic limb and prevent the tendon from mechanical deterioration due to unloading. A similar result was reported in the study by Theis et al., in which it was found that spasticity in children with cerebral palsy did not lead to significantly lower Achilles tendon stiffness than that of typical developing children [26].

Regarding the effect of WBV on tendon stiffness, a previous animal study by Sandhu and colleagues showed that WBV training for five weeks resulted in increased flexor carpi ulnaris tendon stiffness [9]. However, when applied to humans, the effect of WBV on tendon stiffness has been inconsistent [10]. A study by Han et al. showed that eight weeks of WBV training increased Achilles tendon stiffness in older women [11]. Another study, however, revealed no effect on patellar tendon stiffness after eight weeks of WBV training [12]. Regarding the acute effects of WBV on tendon properties, Rieder et al. reported that a single bout of WBV did not change patellar tendon stiffness [13]. In our study, we found no significant changes in the patellar and quadriceps tendon stiffness of both hemiparetic and unaffected limbs immediately after WBV exercise, which was consistent with previous studies [13]. However, we observed that the difference in tendon stiffness between hemiparetic and unaffected limbs, though not reaching statistical significance, was smaller after WBV. This phenomenon could be attributed to the trend of decreasing tendon stiffness after WBV in hemiparetic limbs and relative smaller changes in unaffected limbs. A previous study showed that a single 20 min session of WBV can reduce the spasticity of ankle plantar flexors for 1–2 h in children with cerebral palsy [27]. Another study revealed that a single session of WBV training appeared to successfully reduce ankle spasticity in stroke patients [28]. Therefore, we speculated that a possible mechanism of a trend in the decreased patellar and quadriceps tendon stiffness of hemiparetic limbs is the immediate effect of WBV of decreasing spasticity.

A limitation of this study was its small sample size of enrollment. Moreover, only one session of WBV exercise was performed, and the longitudinal effect was not evaluated. Additionally, we did not evaluate functional performance metrics, such as walking speed or gait pattern, as outcome measures after single bout of WBV exercise. Further long-term investigations of the longitudinal effects of vibration exercise on changes in knee extensor tendon properties and improvements in functional performance are necessary.

## 5. Conclusions

In conclusion, there were no significant differences in the mechanical properties of the knee extensor tendon between hemiparetic and unaffected limbs. However, the tendon stiffness of hemiparetic limbs was positively correlated to knee extensor spasticity. After a single session of WBV exercise, no significant changes in tendon mechanical properties were observed in either hemiparetic or unaffected limb. However, a trend toward decreased tendon stiffness in hemiparetic limbs have been caused by the immediate effect of WBV in reducing spasticity. Further investigations with increased sample sizes are necessary to determine the biomechanical effects of hemiplegic limbs on the knee extensor tendon’s properties and the long-term effects of WBV on the knee extensor tendon’s properties.

## Figures and Tables

**Table 1:** Baseline sociodemographic and clinical characteristics.

Stroke Patients, *n* = 11	Mean ± SD	*n* (%)
Age	53.64 ± 4.37	
Sex		
Male		7 (63.6)
Female		4 (36.4)
Duration from stroke onset (month)	9.91 ± 1.89	
Height (cm)	162.91 ± 2.52	
Weight (kg)	66.57 ± 2.70	
BMI (kg/cm^2^)	25.01 ± 0.63	
Stroke type		
Hemorrhagic		3 (27.3)
Ischemic		8 (72.7)
Hemiparetic side		
Left		5 (45.5)
Right		6 (54.5)
Brunnstrom stage		
III		4 (36.4)
IV		6 (54.5)
V		1 (9.1)
Muscle power (manual muscle test)		
3		2 (18.2)
4		9 (81.8)
Modified Ashworth scale		
1		2 (18.2)
2		8 (72.7)
3		1 (9.1)

**Table 2:** Thickness and stiffness of patellar and quadriceps tendons in hemiparetic and unaffected sides at baseline.

	Unaffected Side	Hemiparetic Side	*p*-Value
	Mean (SD)	Mean (SD)	
Patellar tendon thickness	0.38 (0.06)	0.40 (0.06)	0.450
Quadriceps tendon thickness	0.46 (0.10)	0.46 (0.09)	0.974
Patellar tendon stiffness	4.57 (0.90)	5.16 (1.66)	0.647
Quadriceps tendon stiffness	4.14 (0.86)	4.72 (1.91)	0.953

Standard deviation (SD).

**Table 3:** Correlation between tendon stiffness and knee extensor spasticity in hemiparetic side at baseline.

	MAS, ρ	*p*-Value
Patellar tendon thickness	−0.05	0.892
Quadriceps tendon thickness	−0.35	0.294
Patellar tendon stiffness	0.62	0.044
Quadriceps tendon stiffness	0.62	0.044

Modified Ashworth scale (MAS).

**Table 4:** Change in patellar and quadriceps tendon stiffness after whole body vibration exercise.

	Pre	Post	Post–Pre	*p*-Value
	Mean (SD)	Mean (SD)	Mean (SE)	
Unaffected side				
Patellar tendon stiffness	4.57 (0.84)	4.57 (0.86)	0.00 (0.28)	0.765
Quadriceps tendon stiffness	4.14 (0.86)	4.00 (0.41)	−0.14 (0.12)	0.365
Hemiparetic side				
Patellar tendon stiffness	5.16 (1.66)	4.80 (1.65)	−0.36 (0.30)	0.320
Quadriceps tendon stiffness	4.72 (1.91)	4.34 (0.96)	−0.38 (0.37)	0.278

Standard deviation (SD); standard error (SE).

**Table 5:** Change in tendon stiffness difference between hemiparetic and unaffected sides after whole body vibration exercise.

	Pre (Hemiparetic–Unaffected)	Post (Hemiparetic–Unaffected)	Post–Pre	*p*-Value
	Mean (SE)	Mean (SE)	Mean (SE)	
Patellar tendon stiffness difference	0.59 (0.53)	0.23 (0.43)	−0.36 (0.26)	0.175
Quadriceps tendon stiffness difference	0.58 (0.48)	0.34 (0.29)	−0.24 (0.38)	0.577

Standard error (SE).

**Table 6:** Correlation between hemiparetic side knee extensor spasticity and change in tendon stiffness after whole body vibration exercise.

	Mean (SD)	MAS, ρ	*p*-Value
Patellar tendon stiffness change (post–pre)	−0.36 (0.30)	−0.39	0.230
Quadriceps tendon stiffness change (post–pre)	−0.38 (0.37)	−0.23	0.503

Standard deviation (SD).

**Table 7:** Correlation between hemiparetic side knee extensor spasticity and change in tendon stiffness difference between hemiparetic and unaffected sides after whole body vibration exercise.

	Mean (SD)	MAS, ρ	*p*-Value
Patellar tendon stiffness difference change (post–pre)	0.36 (0.26)	0.38	0.253
Quadriceps tendon stiffness difference change (post–pre)	0.24 (0.38)	0.01	0.987

Standard deviation (SD).

## Data Availability

The data presented in this study are available on request from the corresponding author. The data are not publicly available due to ethical issue.

## References

[1] Maganaris C.N., Reeves N.D., Rittweger J., Sargeant A.J., Jones D.A., Gerrits K., Haan A.D. (2006). Adaptive response of human tendon to paralysis. Muscle Nerve..

[2] Stenroth L., Peltonen J., Cronin N.J., Sipilä S., Finni T. (2012). Age-related differences in Achilles tendon properties and triceps surae muscle architecture in vivo. J. Appl. Physiol..

[3] Magnusson S.P., Kjaer M. (2019). The impact of loading, unloading, ageing and injury on the human tendon. J. Physiol..

[4] Grosset J.F., Breen L., Stewart C.E., Burgess K.E., Onambélé G.L. (2014). Influence of exercise intensity on training-induced tendon mechanical properties changes in older individuals. Age.

[5] Eriksen C.S., Svensson R.B., Gylling A.T., Couppé C., Magnusson S.P., Kjaer M. (2019). Load magnitude affects patellar tendon mechanical properties but not collagen or collagen cross-linking after long-term strength training in older adults. BMC Geriatr..

[6] Hunnicutt J.L., Gregory C.M. (2017). Skeletal muscle changes following stroke: A systematic review and comparison to healthy individuals. Top. Stroke Rehabil..

[7] Prado-Medeiros C.L., Silva M.P., Lessi G.C., Alves M.Z., Tannus A., Lindquist A.R., Salvini T.F. (2012). Muscle atrophy and functional deficits of knee extensors and flexors in people with chronic stroke. Phys. Ther..

[8] Alam M.M., Khan A.A., Farooq M. (2018). Effect of whole-body vibration on neuromuscular performance: A literature review. Work.

[9] Sandhu E., Miles J.D., Dahners L.E., Keller B.V., Weinhold P.S. (2011). Whole body vibration increases area and stiffness of the flexor carpi ulnaris tendon in the rat. J. Biomech..

[10] Fowler B.D., Palombo K.T.M., Feland J.B., Blotter J.D. (2019). Effects of Whole-Body Vibration on Flexibility and Stiffness: A Literature Review. Int. J. Exerc. Sci..

[11] Han S.W., Lee D.Y., Choi D.S., Han B., Kim J.S., Lee H.D. (2017). Asynchronous Alterations of Muscle Force and Tendon Stiffness Following 8 Weeks of Resistance Exercise with Whole-Body Vibration in Older Women. J. Aging Phys. Act..

[12] Rieder F., Wiesinger H.P., Kösters A., Müller E., Seynnes O.R. (2016). Whole-body vibration training induces hypertrophy of the human patellar tendon. Scand. J. Med. Sci Sports.

[13] Rieder F., Wiesinger H.P., Kösters A., Müller E., Seynnes O.R. (2016). Immediate effects of whole body vibration on patellar tendon properties and knee extension torque. Eur. J. Appl. Physiol..

[14] Davis L.C., Baumer T.G., Bey M.J., Holsbeeck M.V. (2019). Clinical utilization of shear wave elastography in the musculoskeletal system. Ultrasonography.

[15] Taş S., Yılmaz S., Onur M.R., Soylu A.R., Altuntaş O., Korkusuz F. (2017). Patellar tendon mechanical properties change with gender, body mass index and quadriceps femoris muscle strength. Acta. Orthop. Traumatol. Turc..

[16] Zardi E.M., Franceschetti E., Giorgi C., Palumbo A., Franceschi F. (2019). Reliability of quantitative point shear-wave ultrasound elastography on vastus medialis muscle and quadriceps and patellar tendons. Med. Ultrason..

[17] Quack V., Betsch M., Hellmann J., Eschweiler J., Schrading S., Gatz M., Rath B., Tingart M., Laubach M., Kuhl C. (2020). Evaluation of Postoperative Changes in Patellar and Quadriceps Tendons after Total Knee Arthroplasty-A Comprehensive Analysis by Shear Wave Elastography, Power Doppler and B-mode Ultrasound. Acad. Radiol..

[18] Zhao H., Ren Y., Wu Y.N., Liu S.Q., Zhang L.Q. (2009). Ultrasonic evaluations of Achilles tendon mechanical properties poststroke. J. Appl. Physiol..

[19] Dias C.P., Freire B., Goulart N.B.A., Castro C.D.D., Lemos F.D.A., Becker J., Arndt A., Vaz M.A. (2019). Impaired mechanical properties of Achilles tendon in spastic stroke survivors: An observational study. Top. Stroke Rehabil..

[20] Svantesson U., Takahashi H., Carlsson U., Danielsson A., Sunnerhagen K.S. (2000). Muscle and tendon stiffness in patients with upper motor neuron lesion following a stroke. Eur. J. Appl. Physiol..

[21] Zhao H., Ren Y., Roth E.J., Harvey R.L., Zhang L.Q. (2015). Concurrent deficits of soleus and gastrocnemius muscle fascicles and Achilles tendon post stroke. J. Appl. Physiol..

[22] Arene N., Hidler J. (2009). Understanding motor impairment in the paretic lower limb after a stroke: A review of the literature. Top. Stroke Rehabil..

[23] Souissi H., Zory R., Bredin J., Roche N., Gerus P. (2018). Co-contraction around the knee and the ankle joints during post-stroke gait. Eur. J. Phys. Rehabil. Med..

[24] Yuan H., Ge P., Du L., Xia Q. (2019). Co-Contraction of Lower Limb Muscles Contributes to Knee Stability During Stance Phase in Hemiplegic Stroke Patients. Med. Sci. Monit..

[25] Akbas T., Kim K., Doyle K., Manella K., Lee R., Spicer P., Knikou M., Sulzer J. (2020). Rectus femoris hyperreflexia contributes to Stiff-Knee gait after stroke. J. Neuroeng. Rehabil..

[26] Theis N., Mohagheghi A.A., Korff T. (2016). Mechanical and material properties of the plantarflexor muscles and Achilles tendon in children with spastic cerebral palsy and typically developing children. J. Biomech..

[27] Park C., Park E.S., Choi J.Y., Cho Y., Rha D.W. (2017). Immediate Effect of a Single Session of Whole Body Vibration on Spasticity in Children With Cerebral Palsy. Ann. Rehabil. Med..

[28] Chan K.S., Liu C.W., Chen T.W., Weng M.C., Huang M.H., Chen C.H. (2012). Effects of a single session of whole body vibration on ankle plantarflexion spasticity and gait performance in patients with chronic stroke: A randomized controlled trial. Clin. Rehabil..

